# Alcohol outlets and alcohol consumption in changing environments: prevalence and changes over time

**DOI:** 10.1186/s13011-021-00430-6

**Published:** 2022-02-04

**Authors:** Amy H. Auchincloss, Saima Niamatullah, Maura Adams, Steven J. Melly, Jingjing Li, Mariana Lazo

**Affiliations:** 1 Department of Epidemiology and Biostatistics, Dornsife School of Public Health, Drexel University, Philadelphia, PA 19104 USA; 2 Urban Health Collaborative, Dornsife School of Public Health, Drexel University, Philadelphia, PA 19104 USA; 3 Community Health and Prevention, Dornsife School of Public Health, Drexel University, Philadelphia, PA 19104 USA

**Keywords:** Alcohol outlets, Alcohol consumption, Public policy, Epidemiology, Public health

## Abstract

**Background:**

To examine whether changes in density of neighborhood alcohol outlets affected changes in alcohol consumption 1-year after regulatory changes increased alcohol availability.

**Methods:**

Person-level data came from a population-based cohort (aged 21–64) residing in/around the Philadelphia, Pennsylvania metropolitan area (2016–2018, *N* = 772). Fifty-eight percent lived in a state that began implementing new regulations (Pennsylvania) and the remainder lived in states without major regulatory changes (Delaware and New Jersey). Alcohol consumption was assessed as days per week (pw), drinks pw, high consumption (≥8 drinks pw), and binge drinking. Availability of off-premise alcohol outlets was assessed using 1-mile density and distance. Regression models adjusted for age, gender, race/ethnicity, income, education, health status, state and population density.

**Results:**

Cross-sectional analyses found that higher outlet density was associated with more alcohol consumption (days, drinks, high consumption; all *p* < 0.03) and residing farther from an outlet was associated with less alcohol consumption (days and drinks; all *p* < 0.04). In longitudinal analyses, relative to no change in outlets, exposure to more outlets was associated with 64% higher odds of drinking on more days pw (p < 0.049) and 55% higher odds of consuming more drinks pw (p < 0.081). However, the longitudinal association between changes in outlets and changes in consumption did not differ for residents in Pennsylvania vs. nearby states. In cross-sectional and longitudinal analyses, outlets were not related to binge drinking.

**Conclusion:**

Off-premise outlets were associated with alcohol consumption consistently in cross-sectional analysis and in some longitudinal analyses. Results can inform future studies that wish to evaluate longer-term changes in increased alcohol availability and effects on consumption.

**Supplementary Information:**

The online version contains supplementary material available at 10.1186/s13011-021-00430-6.

## Background

Research investigating geographic access to alcohol outlets on alcohol consumption posits that having many opportunities for purchasing alcohol makes it convenient to buy alcohol, exposes individuals to alcohol marketing (at point of purchase or outside venues that sell alcohol), and promotes alcohol use as a normative behavior [1, 2]. For these reasons, availability of neighborhood alcohol outlets (defined as higher density of alcohol outlets within geographical areas or closer proximity to outlets) may be associated with alcohol use, higher frequency of alcohol consumption and higher volume of alcohol consumption.

A number of reviews have summarized results from studies that examined whether geographic access to alcohol was associated with higher alcohol consumption among adults [1–3]; findings from these reviews suggest a positive association [1–4]. However, studies have primarily used alcohol sales or household purchases to proxy individuals’ consumption (for example: [5]) and most have been cross-sectional (for example [6]). Two longitudinal studies used middle-aged to older-aged cohorts to study effects of off-premise alcohol availability on alcohol consumption [7, 8]. One Finnish study used a predominantly female middle-aged cohort (mean age of participants was 44.1) and found that, after approximately seven years, risk of heavy alcohol use (roughly equivalent to > 2 drinks per day, the only outcome evaluated) increased when alcohol retail outlets became closer to cohort members’ homes [8]. Increases in outlet density showed expected increases in heavy alcohol use, but results were not statistically significant. Another study in the U.S., used an older cohort of men and women (mean age 62 at baseline) and found – after approximately ten years – density of liquor store outlets (no other types of off-premise outlets were included) was associated with increases in weekly alcohol consumption (but not current alcohol use or heaviest drinking days) [7]. Given the paucity of longitudinal studies in adult samples and the diversity of alcohol contexts studied and measures used, more longitudinal studies are needed in order to gain more insight into this topic.

In the U.S., states and counties set their own regulations that govern retail availability of alcohol. Over the past 50 years, most states have relaxed control over alcohol sales, however, in some states/counties, restrictive regulations remain such as selling alcohol only in state-owned stores with limited hours of operation. Public health advocates generally support restricting alcohol sales including retaining state monopolies over alcohol sales [9]. Monopolies can quite easily set limits on sale hours, retail locations, and minimum prices; whereas private businesses are more likely to push for greater availability and incentivize alcohol purchases via prices and marketing [1, 10–13].

Two research groups have used quasi-experimental studies to test whether privatization of alcohol – meaning, converting from state monopolies over alcohol sales to private business- and subsequent increases in alcohol retail density, impacted residents’ drinking. Results have been mixed. A Canadian research team found that partial privatization of British Columbia’s retail alcohol monopoly 2003–2008 resulted in increased per capita alcohol sales [14]. A U.S. research team found overall per capita liquor sales declined during the two years after implementation of a 2012 regulation that privatized Washington state’s liquor outlets [5, 15]. Besides mixed results, generalization of results from one context to another is very challenging due to diversity of alcohol regulations across states/localities [16] as well as diversity of their commercial and socio-cultural contexts. More evidence is needed to evaluate what effect privatization has on residents’ alcohol consumption.

Pennsylvania is one of the few remaining states in the U.S. where alcohol is not convenient to purchase for off-premise consumption. Until recently, the state only permitted purchase of wine and spirits from retailers at state-owned/operated stores (AKA state-owned retail monopoly) and purchase of beer for off-premise consumption was almost entirely limited to private beer distribution outlets (stores that only sell beer and non-alcoholic beverages. In September 2016, the state started to phase-in new regulations that relaxed the state’s retail monopoly of wine and increased the number of private licenses for beer. For the first time, grocers could apply for a license to sell alcohol (beer and wine). The new regulations included a few other provisions such as extension of Sunday sale hours, relaxing restrictions on alcohol marketing, and allowing beer distributors to sell six-packs and individual bottles and cans (rather than only 24-pack cases).

The current study will describe prevalence and short-term changes in off-premise alcohol outlet density and alcohol use in a Pennsylvania county that is undergoing a relaxation of alcohol sales/availability and surrounding comparisons counties. The comparison counties are in New Jersey and Delaware where there have been private off-premise sales of alcohol for decades and no major changes in alcohol regulations occurred during the study period (Supplement Table 1). The longitudinal hypothesis was that after approximately 1 year of follow-up, increases in alcohol outlets would be associated with increases in alcohol consumption. Further, we tested whether this association was stronger in Pennsylvania (undergoing changes in alcohol regulations) vs. non-Pennsylvania. The added value of this study is the use of longitudinal data during a period where alcohol regulations changed, inclusion of multiple measures of outlets (density and proximity), and multiple measures of alcohol consumption (drinking days and drinks, high number of weekly drinks, and binge drinking).

## Materials and methods

### Data

Person data came from a telephone survey conducted among residents aged 18–64 who lived in Philadelphia, Pennsylvania (“Pennsylvania-group”) and nearby cities in New Jersey and Delaware (Trenton, New Jersey; Camden, New Jersey; and Wilmington, Delaware, “Non-Pennsylvania group”) [17]. The survey was designed to study changes in sugary beverage consumption after implementation of an excise tax on such beverages in Philadelphia but it also included questions on alcohol consumption. Survey participants were recruited in December 2016–February 2017 (baseline period) using a probability-based sampling method to select phone numbers from the target areas (random-digit-dialing). A landline and cell phone dual-frame design was used [18]. Participants were paid $20 at follow-up but were not paid at baseline. The study was approved by Drexel University IRB under expedited review, and verbal informed consent of subjects was obtained by GfK, a professional survey firm contracted for data collection.

Participants who responded to the baseline survey were re-contacted approximately one year later for a follow-up survey (December 2017–March 2018). Alcohol questions were only asked if the participant was legally permitted to drink (aged ≥21). Details on the sample size are provided in section “Analytical sample”.

### Measures

#### Outcomes: alcohol consumption

Participants aged ≥21 were asked if they consumed any type of alcoholic beverage in the past 30 days. Only past 30 days was asked in order to keep the phone survey focused/brief (and has been used by others [19]). If the participant answered ‘yes’ to past 30 day alcohol consumption, then subsequent questions were asked regarding number of days, number of drinks consumed per day, and binge drinking. Binge drinking was assessed using the definition from the U.S. Substance Abuse and Mental Health Services Administration [20]: at least one day in past 30 days the person consumed a high volume of alcohol on a single occasion (≥5 alcoholic drinks for males and ≥ 4 for females). See Supplement Table 2 for additional details on the alcohol measures.

In cross-sectional analyses, alcohol use was operationalized in four ways. We used number of days consumed alcohol in past week (continuous variable), number of alcohol drinks consumed alcohol in past week (continuous variable), high number of drinks per week relative to others in the cohort (top quintile, ≥8 drinks per week, binary variable), and binge in past 30 days (binary variable). The rationale for using within-sample ranking of alcohol consumption is that it acknowledges the low precision inherent in alcohol self-reports [21] and downward recall bias of drinking [22]. Numerous studies have used ranked values to define alcohol consumption (for example [23, 24]) because it differentiates lower and higher values within a sample without relying on an absolute threshold [25].

In longitudinal analyses, within-person change in alcohol consumption was operationalized using the same variables described for cross-sectional analysis. Alcohol at follow-up was subtracted from baseline and then classified into no change (equal to zero), decrease (< 0), increase (> 0). One-year change in alcohol consumption was small thus > 0 was used in order to identify all changes in use.

#### Additional survey measures - covariates

The survey collected socio-demographic data (age, gender, race, education, total annual income for the household from all sources before taxes) as well as select health-related variables (height, weight, smoking, and chronic cardiovascular conditions [high blood pressure, high cholesterol, diabetes, or history of heart disease]). Per capita income was household income divided by number of people in the household supported by the household income. Participant data were imputed if missing race, education, and income per capita (using census ZIP code data [< 5% of participants] or stratified sample means [by age group, gender, and race, < 0.5% of participants]).

#### Exposures: proximity and density of alcohol outlets

Participants’ home locations at baseline and follow up were geocoded to their street address (or to the population weighted centroids if only a ZIP code was reported, 4%) and then assigned alcohol outlet density and proximity measures.

Alcohol outlet data were obtained from state alcohol licensing agencies. We compiled data only for off-premise outlets, defined as where alcohol was purchased for off-premise consumption only (not bars and restaurants). The decision to limit to off-premise outlets was because literature has generally found a stronger associations between off-premise outlets and health outcomes [2, 26, 27].

Proximity was operationalized as the street network (driving) distance between a participant’s residence and the nearest off-premise outlet.

Density was operationalized as the number of off-premise outlets within 1.6 km (1 mile) of participants’ residences. Buffer radius of 1.6 km was used because it represents a 20 minute walk or short drive which has practical relevance for accessing alcohol outlets among both highly urbanized and suburban residents and has been sufficiently sensitive to identify associations between off-premise alcohol and alcohol use in other alcohol studies [6, 28]. Outlet density was further standardized by the population in the buffer: outlet density per 1.6 km buffer, per 10,000 population within buffer. Per 10,000 population was used to improve comparability of residents in Pennsylvania (represented primarily by the county of Philadelphia where population density was high) to residents in non-Pennsylvania counties (a combination of urban and suburban counties).

### Analytical sample

There were 2555 survey participants aged ≥21 who were asked the alcohol questions at baseline, and 802 of these participants responded to the follow-up survey (31% retention). Despite low cohort retention at follow-up, those included vs. lost-to-follow-up were mostly similar to the analytic sample. Among the 802 participants who responded to the follow-up survey, 30 participants were excluded due to missing outcome or exposure data, leaving 772 participants in the cross-sectional analyses. For longitudinal analyses, an additional 58 persons were excluded due to problems with operationalizing their exposure, leaving 714 participants in longitudinal analyses. (See details on the analytic sample in Supplement Fig. 1 and Supplement Table 3.)

### Statistical analysis

Descriptive statistics summarized participant characteristics by alcohol consumption status, state (Pennsylvania, and non-Pennsylvania), at baseline and follow-up.

#### Cross-sectional

Due to skewed distributions and potential non-linear associations with alcohol use, for cross-sectional regression analyses we ranked density of alcohol outlets into quartiles and ranked distance to nearest outlet into tertiles. Tertiles were used for distance (rather than quartiles) in order to improve interpretation (the first quartile category was a very short distance and only applied to the Pennsylvania site, mostly represented by Philadelphia). Tests for linear trend used the ranked categories as ordinal (continuous) variables. Logistic regression (for binary variable high alcohol consumption) and Poisson regression (for count variable, number of drinking days per month and drinks per week) adjusted for age, gender, race/ethnicity, per capita income, educational attainment, history of chronic disease, and population density.

#### Longitudinal

Longitudinal analyses used multinomial logistic regression to model a 3-category outcome: no change (=0), decrease (< 0), increase (> 0) for change in drinking days, number of drinks per week, and binge drinking. A 3-category exposure change variable was classified as 1.6 km density no change (=0), decrease (< 0), increase (> 0). A small number of participants (8%, *N* = 58) experienced a decrease in alcohol outlet density during follow-up. This category was too small to be interpretable as a primary exposure category in regression, thus it was deleted, leaving 714 participants in the longitudinal analyses (772–58 = 714). Longitudinal analyses adjusted for the same variables in cross-sectional analyses plus whether the participant moved out of the baseline ZIP code over the follow-up (binary), residence at follow-up was in Pennsylvania or not (binary), and population density within the 1.6 km buffer at follow-up (quartiles). See footnote in the regression table results for additional details.

#### Testing effects of Pennsylvania’s regulations

In order to test whether changes to Pennsylvania’s regulations changed residents’ consumption, we added a dummy variable representing residence in Pennsylvania at follow-up (vs. non-Pennsylvania). We examined whether changes in alcohol were different for Pennsylvania vs. other areas (main effect for state). In subsequent models, we included an interaction term between this variable and change in outlets over time (no change vs. increase). This tested whether the within-person association between change in outlets and change in consumption (no change, increase, decrease) differed between Pennsylvania and non-Pennsylvania.

#### Sensitivity

Because alcohol use is known to differ by gender [29], it is possible that the association between outlet density and alcohol consumption could differ by gender. In cross-sectional and longitudinal analyses we assessed heterogeneity in results by gender by including an interaction term between the outlet measures and gender.

The main analyses included all cohort members. We assessed whether results changed after limiting the sample to the subset of cohort members who reported past 30 day alcohol use in either/both baseline and follow-up.

Software utilized for geocoding and spatial data processing was ESRI Business Analyst Address Locator (2016) and ArcGIS Pro v. 2.2. Software utilized for statistical analysis was SAS v. 9.4.

## Results

### Descriptive results

Table 1 shows participant sociodemographic characteristics. Fifty-eight percent of the cohort resided in Pennsylvania at baseline and follow-up (Supplement Table 4). Approximately one-half of the sample was aged < 50, female, had not attained a Bachelor’s degree, and was White race. Sixty-three percent had per capita incomes < 35,000 dollars.

**Table 1:** Characteristics of participants, by alcohol consumption (*N* = 772^a^)

			Alcohol Consumption^a^								
	Total		Number of *days per month* consumed alcohol					High number of *drinks per week* relative to others in cohort (top quintile, ≥ 8 drinks per week)			
								No		Yes	
	N	Col %	Mean	SD	Median	P25	P75	N	Row %	N	Row %
Total	772	100%	5.5	7.1	3.0	0	8	644	83%	128	17%
	**N**	**Col %**	**Mean**	**SD**	**Median**	**P25**	**P75**	**N**	**Col %**	**N**	**Col %**
Age Group											
21 to 34	197	26%	5.9	7.0	4	0	8	155	24%	42	33%
35 to 49	218	28%	6.1	7.2	4	0	8	178	28%	40	31%
50 to 64	357	46%	4.9	7.0	2	0	8	311	48%	46	36%
Gender											
Female	402	52%	4.7	6.6	2	0	8	358	56%	44	34%
Male	370	48%	6.3	7.6	4	0	8	286	44%	84	66%
Race											
Black	259	34%	3.7	5.8	1	0	5	228	35%	31	24%
White	419	54%	6.9	7.7	4	0	10	332	52%	87	68%
Other	94	12%	4.2	5.9	2	0	8	84	13%	10	8%
Income per capita											
< $15 k	149	19%	4.6	6.8	2	0	7	127	20%	22	17%
$15 k - < $35 k	337	44%	4.7	6.2	2	0	8	294	46%	43	34%
$35 k - < $50 k	142	18%	6.2	8.0	3	0	8	113	18%	29	23%
$50 k+	144	19%	7.5	7.9	4	0	12	110	17%	34	27%
Education completed											
High school (HS)^b^	225	29%	5.1	7.4	2	0	8	189	29%	36	28%
Tech school/2 years post HS	170	22%	4.2	6.2	2	0	8	140	22%	30	23%
4 year college	192	25%	6.2	7.2	4	0	8	156	24%	36	28%
Graduate school	185	24%	6.4	7.4	4	0	8	159	25%	26	20%
Chronic conditions, n (%)^c^	313	41%	4.9	7.0	2	0	8	275	43%	38	30%
Moved^d^											
Yes	95	12%	6.2	7.0	4	0	8	70	11%	25	20%
No	677	88%	5.4	7.1	3	0	8	574	89%	103	80%
State (at follow-up)											
Pennsylvania	444	58%	5.1	6.6	3	0	8	370	57%	74	58%
Delaware	148	19%	6.9	8.0	4	1	10	116	18%	32	25%
New Jersey	180	23%	5.4	7.3	2	0	8	158	25%	22	17%

*Abbreviations*: *SD* standard deviation, *P25* 25th percentile, *P75* 75th percentile, *Col* column

^a^Includes participants who did not consume alcohol in past 30 days (*N* = 207 or 27% of the cohort)

^b^Completed high school includes receiving a high school equivalency diploma (GED®)

^c^Presence of a chronic cardiovascular conditions was assessed by asking whether the participant was ever told by a doctor, nurse, or other health professional that they had at least one of the following: high blood pressure, high cholesterol, diabetes, or history of heart disease

^d^Moved out of baseline ZIP code but stayed within study area

#### Alcohol consumption at baseline and follow-up

Twenty-seven percent (*N* = 207) of the cohort did not consume any alcohol at baseline or 1-year follow-up and approximately 10% consumed alcohol only at baseline or follow-up (not both periods).

Table 1 presents the distribution of alcohol use at baseline across socio-demographic characteristics and the Supplement Table 4 presents details on various alcohol measures at baseline and follow-up.

In the full sample, participants who were younger, male, White race, and higher socio-economic status consumed alcohol more frequently than other members of the cohort (median 4 days per month vs. approximately 2 days for others); and were over-represented in the higher consumption group (≥8 drinks or more per week, Table 1).

Among a subset of the cohort who consumed alcohol in either time period (*N* = 565, 73%), median alcohol consumption per week was 1.25 days (25th percentile and 75th percentile [Q1Q3] 0.5, 2.5) and 2.5 drinks (Q1Q3 0.75, 6.0) (not shown in the tables). Among this subset, 22% consumed 8 or more drinks per week and 37% reported at least 1 binge occasion in the past 30 days. Most drinkers reported consuming beer and/or wine (90%, alcoholic beverages that were subject to changes in Pennsylvania alcohol regulations), 10% reported only drinking spirits (data not shown in tables).

Within-person unadjusted analyses of change found that -- for most of the cohort -- alcohol use was unchanged between baseline and follow-up (days per week median 0 [Q1Q3: − 0.5, 0.24]; drinks per week median 0 [Q1Q3: -0.75, 1] (Table 2). Most participants did not change their binge occasions (68% had no change).

**Table 2:** Unadjusted within-person change in alcohol consumption and alcohol outlets; by total (all), Pennsylvania and non-Pennsylvania, *N* = 772^a^

	All			Pennsylvania			Non-Pennsylvania		
	*N* = 772			*N* = 444			*N* = 328		
**Within-person change in alcohol consumption - change in days, drinks, binge occasions**									
	Median	P25	P75	Median	P25	P75	Median	P25	P75
Number of *days* consumed alcohol per week	0	−0.5	0.25	0	−0.25	0.25	0	−0.5	0.25
	N	%		N	%		N	%	
No change, N (%)	312	40%		195	44%		117	36%	
Decreased, N (%)	238	31%		119	27%		119	36%	
Increased, N (%)	222	29%		130	29%		92	28%	
	Median	P25	P75	Median	P25	P75	Median	P25	P75
Number of *drinks* per week	0	−0.75	1	0	−0.5	1	0	−1.25	1
	N	%		N	%		N	%	
No change, N (%)	259	34%		161	36%		98	30%	
Decreased, N (%)	252	33%		131	30%		121	37%	
Increased, N (%)	261	34%		152	34%		109	33%	
	Median	P25	P75	Median	P25	P75	Median	P25	P75
Number of *binge* occasions, past 30 days	0	0	0	0	0	0	0	0	0
	N	%		N	%		N	%	
No change, N (%)	522	68%		302	68%		220	67%	
Decreased, N (%)	111	14%		60	14%		51	16%	
Increased, N (%)	139	18%		82	18%		57	17%	
**Within person change in number of off-premise outlets, change in number within buffer**									
	Median	P25	P75	Median	P25	P75	Median	P25	P75
1600 m buffer	0	0	1	0	0	1	0	0	0
	N	%		N	%		N	%	
No change, N (%)	508	66%		281	63%		227	69%	
Decreased, N (%)	58	8%		32	7%		26	8%	
Increased, N (%)	206	27%		131	30%		75	23%	

*Abbreviations*: *SD* standard deviation, *P25* 25th percentile, *P75* 75th percentile, *Col* column

^a^Includes participants who did not consume alcohol in past 30 days in both waves: *N* = 207 or 27% of the cohort

Over the 12-months of follow-up, differences in prevalence of alcohol use across geographical sites narrowed. The prevalence of alcohol use increased slightly in Pennsylvania (61 to 63%) and decreased slightly in non-Pennsylvania (70 to 66%) (Supplement Table 4). However, among alcohol users, median days consumed alcohol per month remained roughly equivalent across the study sites (4 to 5 days per month).

#### Density and proximity of alcohol outlets at baseline and follow-up

Pennsylvania was represented primarily by Philadelphia which is a large urban area with higher population density than non-Pennsylvania areas. For example, Philadelphia, median density at follow-up was approximately 46,800 population in a1.6 km buffer which translates to 5818 per square km or 15,070 per square mile. Median density in non-Pennsylvania areas was approximately 10,000 in a 1.6 km buffer which translates to 1243 per square km or 3220 per square mile; see Supplement Table 4). Population density was correlated with outlet density (Spearman’s rank correlation coefficient = 0.84). At baseline, Pennsylvania residents lived in areas with higher density of outlets than non-Pennsylvania residents and were much closer to an outlet. For example, median number of off-premise outlets within 1.6 km buffer for participants in Pennsylvania was 7 (25th–75th percentile [Q1Q3]: 3–13) vs. in non-Pennsylvania median was 2 (Q1Q3: 0–5); median distance to the nearest outlet was 742 m in Pennsylvania vs. 1205 m in non-Pennsylvania. More than one-half of the sample experienced no change in the density of off-premise outlets (66% for all). The proportion of the cohort that experienced an increase was higher in Pennsylvania (30%) than in non-Pennsylvania (23%).

### Adjusted cross-sectional results

Table 3 displays the adjusted cross-sectional results from logistic regression (for binary variable high alcohol consumption) and Poisson regression (for count variable, number of drinking days per month and drinks per week).

**Table 3:** Adjusted^a^ cross-sectional estimates of alcohol consumption with density of off-premise outlets and distance to outlets. *N* = 772

			Number of drinking *days* per week^b^				Number of *drinks* per week				High number of drinks per week relative to others in cohort^c^				Binge at least once in past 30 days^c^			
			(continuous counts)				(continuous counts)				(binary variable)				(binary variable)			
			Exp	95% CI		P	Exp	95% CI		P	Odds	95% CI		P	Odds	95% CI		P
Exposure			(Beta)^c^	low	high	value	(Beta)^c^	low	high	value	ratio	low	high	value	ratio	low	high	value
**Alcohol outlet density in 1.6 km buffer, per 10,000 population**																		
Quartiles^d^																		
Q1.	Lowest	0.0–0.99	Referent				Referent				Referent				Referent			
Q2.		1.0–1.70	0.98	0.82	1.19	0.864	1.02	0.91	1.14	0.728	1.22	0.65	2.32	0.536	1.03	0.64	1.66	0.905
Q3.		1.71–2.8	1.11	0.93	1.33	0.253	1.21	1.09	1.35	0.001	1.97	1.08	3.62	0.028	0.90	0.56	1.45	0.662
Q4.	Highest	2.9–10.7	1.28	1.08	1.52	0.005	1.34	1.21	1.49	<.0001	1.59	0.87	2.92	0.135	1.19	0.75	1.89	0.464
**Distance from participant to nearest off-premise outlet**																		
Tertiles, kilometers^e^																		
T1.	Nearest	0.021–0.622	Referent				Referent				Referent				Referent			
T2.		0.623–1.26	1.00	0.86	1.16	0.993	0.96	0.88	1.05	0.386	0.92	0.56	1.51	0.737	1.09	0.72	1.65	0.674
T3.	Farthest	1.27–10.16	0.81	0.67	0.99	0.038	0.79	0.70	0.88	<.0001	0.58	0.30	1.11	0.100	1.28	0.77	2.12	0.346

*Abbreviations*: *CI* confidence interval

^a^Cross-sectional results follow-up, adjusted for age, gender, race/ethnicity, per capita income, educational attainment, history of chronic disease (binary), state. When per-population was not part of the exposure measure, then the model also adjusted for population density within a 1.6 km area (operationalized into quartiles)

^b^Poisson regression was used to derive these estimates. Beta coefficients represents the difference in the logs of expected drinking days (per week) for discrete exposure category vs. referent category. Exponentiated beta coefficient represents a relative value. Thus, in cross-sectional data the exp.(beta) 1.28 can be interpreted as 28% higher drinking days per month when living in the highest quartile of outlet density (0.29–1.7 per 10,000 population) relative to the lowest quartile (the referent group)

^c^Logistic regression was used to derived these estimates. High number of drinks refers to high consumption relative to others in cohort (top quintile > = 8 drinks per week). Binge in the past 30 days refers to > = 1 time in past 30 days consumed a large volume of alcohol during a single occasion (> = 5 drinks for males, > = 4 drinks for females). For 5 participants, their baseline binge value was used because their follow-up value was missing

^d^The following information attempts to aide interpretation of the quartile groups for alcohol outlet density in a 1.6 km buffer, per 10,000 population. Within each quartile of the standardized count, the median (and P25, P75) of the unstandardized 1.6 km density is as follows: Quartile 1: median 0 outlets (0–1); Quartile 2: median 6 outletS (2, 8), Quartile 3: median 7 outlets (3, 13); Quartile 4: median 11 outlets (4, 25)

^e^Tertitle distances in miles: T1. 0.01–0.386 miles, T2. 0.387–0.78 miles, T3. 0.79–6.31 miles

**Outlet density.** Residents living in areas with higher outlet density areas (quartile 3) did not increase their d*rinking days* however they had 21% more *drinks* per week (expβ = 1.21, 95% confidence interval [CI]: 1.09, 1.35 [*p* < 0.001]). Residents living in the highest outlet density areas (quartile 4) had 28% more *drinking days* per week and 34% more *drinks* per week (outlet density quartile 4 vs. 1, drinking days: expβ = 1.28, 95% CI: 1.08, 1.52 [*p* = 0.005]; drinks per week: expβ = 1.34, 95% CI: 1.21, 1.49 [*p* < 0.001]). The odds of having a *high number of drinks* per week (≥8 drinks per week), was 97% higher for residents living in higher density areas (outlet quartile 3 vs. 1, odds ratio [OR] 1.97, 95% CI: 1.08, 3.62 [*p* = 0.028]) and results suggested an expected positive direction [30] for residents living in the highest density areas quartile 4 vs. 1 (OR 1.59, 95% CI: 0.87, 2.92 [*p* = 0.13]). All tests for linear trend (AKA trend) between outlet density quartiles and drinking outcomes were *p* < 0.002, with the exception of high number of drinking *days* which was *p* < 0.056.

**Outlet distance**. In the adjusted analyses, residents living in areas farthest from an alcohol outlet (tertile 3 vs. 1) had approximately 20% fewer drinking *days* per week (*p* < 0.038, trend was *p* < 0.06) and *drinks* per week (*p* < 0.0001, trend was *p* < 0.0002). Results also suggested an expected negative direction [30] for having a *high number of drinks* per week (≥8 drinks per week) with increases in distance to an alcohol outlet (farther distance to an alcohol outlet tertile 3 vs. 1, OR 0.58, 95% CI: 0.30, 1.11 [*p* < 0.1] and trend *p* < 0.13).

Binge drinking was not associated with alcohol outlet density or proximity.

### Adjusted longtudinal results

Table 4 shows the adjusted multinomial longitudinal results for increases in outlets (vs. no change in outlet density) and change in alcohol consumption. Relative to no change in alcohol outlet density, residents exposed to increases in alcohol outlet density had 64% higher odds of increasing their *drinking days* per week (compared to those with no change in consumption, OR: 1.64, 95% CI: 1.00, 2.67 [*p* < 0.049]) and results also suggested *drinks per week* increased (note that most of the confidence interval is above the null value, which aligned with an expected positive direction [30] OR: 1.55, 95% CI: 0.95, 2.55 [*p* < 0.081]). Point estimates were positive for increases in outlet density and decreases in drinking days per week (vs. no change in consumption); however – compared to results for increases in alcohol consumption -- odds ratios were weaker and *p* values higher (decrease in drinking days per week *p* < 0.41; decrease in number of drinks per week *p* < 0.91).

**Table 4:** Multinomial regression results. Adjusted^**a**^ within-person change in alcohol consumption (change in days, drinks, binge occasions) for an increase in off-premise alcohol outlets within a 1.6 km buffer^**b**^. *N* = 714^**c**^

		Distribution	Adjusted	95% confidence interval		P
	N	in the sample	odds ratio^d^	low	high	value
Change in number of drinking *days* per week						
1. No change	288	40%	Referent			
2. Increased	206	29%	1.64	1.00	2.68	0.049
3. Decreased	220	31%	1.23	0.76	2.00	0.409
Change in number of *drinks* per week						
1. No change	239	33%	Referent			
2. Increased	244	34%	1.55	0.95	2.55	0.081
3. Decreased	231	32%	0.97	0.58	1.62	0.908
Change number of *binge* occasions (past 30 days)^**e**^						
1. No change	483	68%	Referent			
2. Increased	126	18%	1.16	0.68	1.97	0.588
3. Decreased	105	15%	1.01	0.57	1.79	0.984

*Abbreviations*: *CI* confidence interval

^a^Adjusted for age at baseline, gender, race/ethnicity, per capita income, education, history of chronic disease (binary), moved from ZIP code at follow-up, state at follow-up (Pennsylvania vs non-Pennsylvania), and population density per 1.6 km area (quartiles)

^b^The exposure is a binary variable: increase in outlets vs. no increase in outlets (referent category) using the measure ‘count of outlets in 1.6 km buffer’. The category for ‘decrease’ in outlets was not included because very few participants experienced a decrease in outlets. Per population standardization was not needed for the exposure variable in longitudinal model because the exposure was within-person change in outlet exposure and population density did not change much (because participants remained in their state). Nevertheless, we included population density (quartiles) as an adjustment variable in the model

^c^The sample size for this table slightly decreased (from *N* = 772 to *N* = 714). We deleted the 58 participants who experienced a decrease in alcohol outlet density during follow-up

^d^Odds ratios derived from multinomial logit regression as appropriate for 3-level outcome: alcohol consumption no change (referent category), decrease, increase. Change defined as | > 0| days per week, | > 0| drinks per week, | > 0| binge days per month

^e^Binge refers to past 30 days consumed a large volume of alcohol during a single occasion (> = 5 drinks for males, > = 4 drinks for females)

There was no evidence that change in outlets was related to an increase or decrease in past 30 days binge drinking (*p* > 0.6).

### Interactions

 We found no strong evidence that there were cross-sectional differences by gender in the association between alcohol outlets (density or proximity) and drinking days or high alcohol use or binge in past 30 days (*p* for interaction > 0.09 and no evidence from longitudinal analyses that there were differences by gender sex in the association between change in outlet and change in consumption (*p* for interaction > 0.18). The exception to this was in cross-sectional analysis, the association between alcohol outlets (density or proximity) and drinks per week was stronger for males than females (*p* for interaction < 0.0001, Supplement Fig. 2).

In longitudinal analyses, before inclusion of an interaction term, we examined the main effect of state (living in Pennsylvania vs outside Pennsylvania). There were no differences by state at follow-up in changes in drinking (*p* > 0.4) except that residents in Pennsylvania, had lower adjusted odds of decreasing their drinking (days per week [β − 0.0280, *p* < 0.005] and drinks per week [β − 0.5302, *p* < 0.037], not shown).

In longitudinal analysis after including an interaction between state and changes in alcohol outlets, there was no evidence that the association between changes in outlet density on changes in alcohol consumption differed for residents in Pennsylvania (where new regulations were being phased-in) vs. other areas (p for interaction > 0.6 for outcomes drinks per day and drinks per week).

### Sensitivity: subset sample to past 30 day alcohol users

When the sample was subset to only cohort members who reported past 30 day use of alcohol, cross-sectional inference was largely the same (Supplement Table 5). Longitudinal analyses showed a positive point estimate for increases in outlet density and increase in drinking days per week (vs. no change in consumption), but the *p* value was not statistically significant (likely due to insufficient contrast in outcome and decreased sample size) (Supplement Table 6).

## Discussion

This population-based study described prevalence and short-term changes in off-premise alcohol outlets and alcohol use in a Pennsylvania county where new regulations were being phased-in vs. counties in surrounding states where no major changes in outlet licensing occurred. Cross-sectional results adjusted for confounders, consistently found residents living in higher outlet density areas and closer to outlets consumed more alcohol (weekly number of days consumed alcohol and number of drinks per week). Longitudinal analyses of within-person changes over one year follow-up found that residents exposed to increased alcohol outlet density had increases in the number of drinking days and drinks per week. In cross-sectional and longitudinal analyses there was no association for binge drinking.

Our cross-sectional results found that drinking days per week was 28% higher and drinks per week was 34% higher for residents in areas with the highest density of outlets vs. lowest density. Using the prevalence of alcohol consumption in our sample, this translates to an average of approximately 3 more drinking days per month or -- among more frequent alcohol users (those in the top quartile of drinks per week) -- approximately 8 more drinking days per month. In general, our results aligned with positive associations between outlet density and/or proximity and alcohol consumption found in other work and summarized in reviews [1–3], however, most prior work proxied individual alcohol consumption via aggregate sales or purchases [5].

Our longitudinal results found that relative to no change in alcohol outlet density and no change in consumption, residents exposed to increases in alcohol outlet density had 64% higher drinking days per week. Only two previous cohort studies examined changes among adults in alcohol consumption in response to changes in off-premise outlets. One of the studies [7], followed a middle-aged/older cohort for 10 years who resided in diverse regions in the U.S.. Results can roughly be translated as follows: for an increase in 4 liquor stores within a 1-mile area, drinks per week increased by roughly 10%; liquor stores were the only type of outlets included. The other study [8], followed a middle-aged cohort in Finland for 7 years and only evaluated the risk of heavy alcohol use (roughly equivalent to at least 2 or 3 drinks per day, approximately 10% of the cohort). Halonen et al. found risk of heavy alcohol use increased with geographic access to outlets [8]. In contrast, longitudinal results from our study and from the other U.S. study did not find associations between change in outlets and changes in heavy alcohol use. Differences in context/regulations, measurement, and cohort composition (for example, the Finnish study was a cohort of public sector employees where heavier drinking was more prevalent) make the studies difficult to compare.

We tested whether the longitudinal associations were stronger among participants who resided in Pennsylvania (where changes in alcohol regulations were underway) vs. those from non-Pennsylvania. We found no evidence of this: within-person change in outlets and within person change in alcohol consumption did not differ for Pennsylvania vs. non-Pennsylvania. Our ability to detect differences by state was constrained because over one year, changes in alcohol outlets were small. Although the proportion of the cohort that experienced an increase was somewhat higher in Pennsylvania (30%) than in non-Pennsylvania (23%), most of the cohort’s within-person change in outlet exposure was due to relocation within the study area rather than within-neighborhood change in outlets. During the study period, changes in Pennsylvania’s alcohol regulations were being phased-in [31]. Approximately every few months, the state licensing agency made additional licenses available and businesses could apply for them and then compete in a license lottery. 

Results assessing exposure to outlets and alcohol consumption are difficult to generalize across diverse alcohol regulatory environments, and socio-cultural differences by region. For this reason, it is important to continue to conduct regional studies on this topic [2]. Two research groups have used quasi-experiments to test whether privatization of alcohol, and subsequent increases in alcohol retail density, impacted residents’ drinking measured via purchases of alcohol (or retail sales). Both research groups found increases in outlets post- privatization. Additionally, the Canadian team found increases in per capita alcohol sales approximately 5 years post-privatization [14] while the US team found overall sales declined approximately two to three years post-privatization [5, 15] although alcohol purchases increased among some low and moderate alcohol purchasers [15]. Possible reasons for the decline in sales in Washington state was that privatization was accompanied by increase in prices [32].

Given well-documented secular increases in alcohol consumption among women [29, 33], examination of gender-differences in associations between neighborhood outlets and drinking is an important area for future research. Our study mostly did not detect differences in the association between outlets and alcohol consumption by gender. The exception was cross-sectional associations between outlets and drinks per week which was stronger for males; however, there were no gender-differences in our longitudinal analyses. The only other U.S. adult cohort study that is comparable to our study found stronger longitudinal associations for men (compared to women) [7]. If gender-differences in the association between neighborhood alcohol outlets and drinking are true, we conjecture that perhaps gender-differences in food/beverage shopping account for some of the variation [34,35].

### Summary of study strengths and limitations

Distinct strengths of this study are *1.* We leveraged changes in alcohol environment to conduct cross-sectional and longitudinal analyses examining the association between alcohol outlets density and proximity and self-reported alcohol consumption. *2.* We geocoded alcohol license data and created multiple measures of access: density in the 1-mile area surrounding the participant’s residence and a proximity measure that measured distance using the street network. This is an improvement on other work that assessed access to outlets using an administrative unit such as census tracts [36, 37] or self-reported access to outlets [38] and work that only focused on either density or distance but not both [7]. *3.* Compared to prior studies using alcohol sales information, we included multiple measures of alcohol consumption (drinking days and drinks, high number of weekly drinks, and high volume of alcohol consumed in a single occasion).

There are a few limitations worth noting. *1.* The limitations of alcohol consumption data are well-known, particularly regarding under-reporting among adults [39]. Nevertheless, the sample distribution for alcohol consumption in our dataset roughly aligned with distributions reported in national surveillance datasets [29, 40] – although our estimates were slightly higher. For example, surveillance data reports that 55% of adults aged ≥18 reported drinking in the past month [29] and in our data it was approximately 65% among aged 21 or older. Surveillance data reported approximately 25% of aged ≥25 reported binge drinking in past month (defined as 4 drinks females/5 drinks males per occasion in past month) [40] and in our data approximately 30% reported at least one binge day in past month. *2.* Some of the analyses were cross-sectional which are subject to temporal biases. Further, there was only 1-year between baseline and follow-up for the longitudinal period which limited our methods and inference for longitudinal analyses. There were generally only small changes in alcohol consumption and alcohol outlet exposures over this period, thus hampering our ability to detect hypothesized signals from the longitudinal data. *3.* Our survey did not ask alcohol consumption questions to participants who were not legally able to purchase alcohol (aged < 21) thus it is unknown if results would generalize to them.

## Conclusions

This study – conducted in a context where alcohol regulations were undergoing liberalization – affirmed that off-premise outlet density and proximity were positively associated with alcohol consumption in cross-sectional analyses. In longitudinal analyses, our findings suggested that increases in outlet density may promote more alcohol consumption; results were strongest for frequency of days consumed alcohol.

In most areas of the world, alcohol availability and marketing are increasing as is alcohol use [41]. Public health advocates generally support restricting alcohol sales including maintaining state monopolies over alcohol sales. Post-privatization, the number of alcohol outlets tend to increase dramatically leading to much greater access/availability of alcohol [1, 11]. Increased competition among retailers leads to dramatic increases in alcohol advertising [12] and the rapid rise in outlets combined with less government oversight can lead to decreased enforcement of minimum-drinking-age laws and other sales restrictions [13].

In North America, the general public continues to broadly support greater access to alcohol [42] and has low understanding of the connections between availability of alcohol in food/beverage stores and harmful alcohol use in adult populations [43, 44]. The alcohol industry’s expansive marketing activities are difficult to counter [45]. Nevertheless, it is important that alcohol control programs use the available research evidence to better-communicate how liberalization of alcohol outlet regulations can promote alcohol consumption and the personal and community-level harms from alcohol use [16]. More longitudinal studies are needed in order to build the evidence base – particularly in areas considering policy changes. Results from this study can inform future studies that wish to evaluate longer-term relaxation of alcohol control that could affect alcohol availability and alcohol consumption.

## Supplementary Information

**Additional file 1. **Supplement to main text description of the analytical sample. **Supplement Fig. 1.** Enrollment and retention. **Supplement Table 1.** Baseline policies related to off-premise sale of alcohol in Pennsylvania and the two comparison states (New Jersey and Delaware). **Supplement Table 2.** Additional details on alcohol consumption measures. **Supplement Table 3.** Characteristics of included vs. excluded participants. **Supplement Table 4.** Alcohol consumption and alcohol outlets, baseline and the one-year follow-up for total, Pennsylvania and non-Pennsylvania, *N* = 772. **Supplement Table 5.** Sensitivity analysis - sample subset to drinkers. Adjusted cross-sectional estimates of alcohol consumption with density of off-premise outlets and distance to outlets. *N* = 565. **Supplement Table 6.** Sensitivity analysis - sample subset to drinkers. Multinomial regression, adjusted within-person change in alcohol consumption (change in days, drinks, binge occasions) for an increase in off-premise alcohol outlets within a 1.6 km buffer (> 0 increase in outlets). *N* = 521. **Supplement Fig. 2** Adjusted cross-sectional estimates of exposure to off premise alcohol outlets (density of and distance to outlets) and number of drinks per day, stratified by gender.

## Data Availability

This study’s data are not publicly available due to human subjects research protections but qualified academic researchers may send data requests to the corresponding author for review. All use of the data would be subject to confidentiality and data-use agreements.

## Abbreviations

CI: Confidence interval
KM: Kilometer
Q1Q3: 25th percentile and 75th percentile
OR: Odds ratio
expβ: Exponentiated beta coefficient

## References

[1] Campbell CA, Hahn RA, Elder R, Brewer R, Chattopadhyay S, Fielding J, Naimi TS, Toomey T, Lawrence B, Middleton JC, Task Force on Community Preventive Services (2009). The effectiveness of limiting alcohol outlet density as a means of reducing excessive alcohol consumption and alcohol-related harms. Am J Prev Med.

[2] Gmel G, Holmes J, Studer J (2016). REVIEW are alcohol outlet densities strongly associated with alcohol-related outcomes? A critical review of recent evidence. Drug Alcohol Rev..

[3] Popova S, Giesbrecht N, Bekmuradov D, Patra J (2009). REVIEW hours and days of sale and density of alcohol outlets: impacts on alcohol consumption and damage: a systematic review. Alcohol Alcohol.

[4] Sherk A, Stockwell T, Chikritzhs T, Andréasson S, Angus C, Gripenberg J, Holder H, Holmes J, Mäkelä P, Mills M, Norström T, Ramstedt M, Woods J (2018). Alcohol consumption and the physical availability of take-away alcohol: systematic reviews and meta-analyses of the days and hours of sale and outlet density. J Stud Alcohol Drugs.

[5] Ye Y, Kerr WC (2016). Estimated increase in cross-border purchases by Washington residents following liquor privatization and implications for alcohol consumption trends. Addiction.

[6] Schonlau M, Scribner R, Farley TA, Theall K, Bluthenthal RN, Scott M (2008). Alcohol outlet density and alcohol consumption in Los Angeles county and southern Louisiana. Geospat Health.

[7] Brenner AB, Borrell LN, Barrientos-Gutierrez T, Diez Roux AV (2015). Longitudinal associations of neighborhood socioeconomic characteristics and alcohol availability on drinking: results from the multi-ethnic study of atherosclerosis (MESA). Soc Sci Med.

[8] Halonen JI, Kivimäki M, Virtanen M, Pentti J, Subramanian SV, Kawachi I, Vahtera J (2013). Proximity of off-premise alcohol outlets and heavy alcohol consumption: a cohort study. Drug Alcohol Depend.

[9] Hahn RA, Middleton JC, Elder R, Brewer R, Fielding J, Naimi TS, Toomey TL, Chattopadhyay S, Lawrence B, Campbell CA, Community Preventive Services Task Force (2012). Effects of alcohol retail privatization on excessive alcohol consumption and related harms: a community guide systematic review. Am J Prev Med.

[10] Room R, Cisneros OJ (2019). Government monopoly as an instrument for public health and welfare: lessons for cannabis from experience with alcohol monopolies. Int J Drug Policy.

[11] Grubesic TH, Murray AT, Pridemore WA, Tabb LP, Liu Y, Wei R (2012). Alcohol beverage control, privatization and the geographic distribution of alcohol outlets. BMC Public Health.

[12] Her M, Giesbrecht N, Room R, Rehm J (1999). Privatizing alcohol sales and alcohol consumption: evidence and implications. Addiction.

[13] Gruenewald PJ (2011). Regulating availability: how access to alcohol affects drinking and problems in youth and adults. Alcohol Res Health.

[14] Stockwell T, Zhao J, Macdonald S, Pakula B, Gruenewald P, Holder H (2009). Changes in per capita alcohol sales during the partial privatization of British Columbia's retail alcohol monopoly 2003-2008: a multi-level local area analysis. Addiction.

[15] Barnett SBL, Coe NB, Harris JR, Basu A (2020). Washington's privatization of liquor: effects on household alcohol purchases from initiative 1183. Addiction.

[16] Blanchette JG, Lira MC, Heeren TC, Naimi TS (2020). Alcohol policies in U.S. states, 1999-2018. J Stud Alcohol Drugs..

[17] Zhong Y, Auchincloss AH, Lee BK, Kanter GP (2018). The short-term impacts of the Philadelphia beverage tax on beverage consumption. Am J Prev Med.

[18] Fahimi M (2014). Practical guidelines for dual-frame RDD survey methodology (now that the dust is settling). Surv Pract.

[19] CDC. 2019 BRFSS Questionnaire https://www.cdc.gov/brfss/questionnaires/pdf-ques/2019-BRFSS-Questionnaire-508.pdf (Last accessed March 20, 2021). 2019.

[20] SAMHSA. Binge Drinking: Terminology and Patterns of Use, 2016. Available at: https://www.samhsa.gov/data/report/2017-methodological-summary-and-definitions. Last accessed 18 Jan 2017.: Substance Abuse and Mental Health Services Administration, U.S. Department of Health and Human Services. ; 2017.

[21] NCI. Food Frequency Questionnaire at a Glance. Available at https://dietassessmentprimer.cancer.gov/profiles/questionnaire/. Last accessed 22 Dec 2016.: National Cancer Institute; 2016.

[22] Ye Y, Bond JC, Cherpitel CJ, Borges G, Monteiro M, Vallance K (2013). Evaluating recall bias in a case-crossover design estimating risk of injury related to alcohol: data from six countries. Drug Alcohol Rev.

[23] Gupta S, Wang F, Holly EA, Bracci PM (2010). Risk of pancreatic cancer by alcohol dose, duration, and pattern of consumption, including binge drinking: a population-based study. Cancer Causes Control.

[24] Schabath MB, Thompson ZJ, Egan KM, Torres BN, Nguyen A, Papenfuss MR, Abrahamsen ME, Giuliano AR (2015). Alcohol consumption and prevalence of human papillomavirus (HPV) infection among US men in the HPV in men (HIM) study. Sex Transm Infect.

[25] Liese AD, Krebs-Smith SM, Subar AF, George SM, Harmon BE, Neuhouser ML, Boushey CJ, Schap TRE, Reedy J (2015). The dietary patterns methods project: synthesis of findings across cohorts and relevance to dietary guidance. J Nutr.

[26] Grubesic TH, Pridemore WA, Williams DA, Philip-Tabb L (2013). Alcohol outlet density and violence: the role of risky retailers and alcohol-related expenditures. Alcohol Alcohol.

[27] Jennings JM, Milam AJ, Greiner A, Furr-Holden CD, Curriero FC, Thornton RJ (2014). Neighborhood alcohol outlets and the association with violent crime in one mid-Atlantic City [Baltimore]: the implications for zoning policy. J Urban Health.

[28] Brenner AB, Diez Roux AV, Barrientos-Gutierrez T, Borrell LN (2015). Associations of alcohol availability and neighborhood socioeconomic characteristics with drinking: cross-sectional results from the multi-ethnic study of atherosclerosis (MESA). Subst Use Misuse.

[29] NIAAA. Alcohol Facts and Statistics https://www.niaaa.nih.gov/publications/brochures-and-fact-sheets/alcohol-facts-and-statistics (Last access 1 Feb 2021). U.S. Department of Health and Human Services, National Institute on Alcohol Abuse and Alcoholism; 2020.

[30] Szklo M, Nieto FJ (2019). Chapter 9. Communicating results of epidemiologic studies. Epidemiology: beyond the basics (shmuacir/file/download/news/1564213580-epidemiology-beyodpdf) [internet].

[31] PLCB. Act 39. HB1690 Liquor Reform Bill http://www.legis.state.pa.us/cfdocs/billInfo/billInfo.cfm?sYear=2015&sInd=0&body=H&type=B&bn=1690. Pennsylvania Liquor Control Board; 2016. Accessed 5 Jan 2022.

[32] Kerr WC, Williams E, Greenfield TK (2015). Analysis of Price changes in Washington following the 2012 liquor privatization. Alcohol Alcohol.

[33] McKetta SC, Keyes KM (2020). Trends in U.S. women's binge drinking in middle adulthood by socioeconomic status, 2006–2018. Drug Alcohol Depend.

[34] Moore LV, Diez Roux AV (2006). Associations of neighborhood characteristics with the location and type of food stores. Am J Public Health.

[35] TrendSource (2018). Consumers’ convenience store purchase frequency in the United States as of 2018, by gender https://www.statista.com/statistics/858176/convenience-store-purchase-frequency-by-gender-us/Grocery (Last accessed 28 Mar 2021).

[36] Ayuka F, Barnett R. Place effects on alcohol consumption: a literature review. J Addict Res Ther. 2015;2015:1–12.

[37] Romley JA, Cohen D, Ringel J, Sturm R (2007). Alcohol and environmental justice: the density of liquor stores and bars in urban neighborhoods in the United States. J Stud Alcohol Drugs.

[38] Pollack CE, Cubbin C, Ahn D, Winkleby M (2005). Neighbourhood deprivation and alcohol consumption: does the availability of alcohol play a role?. Int J Epidemiol.

[39] Stockwell T, Zhao J, Macdonald S (2014). Who under-reports their alcohol consumption in telephone surveys and by how much? An application of the ‘yesterday method’ in a national Canadian substance use survey. Addiction.

[40] SAMHSA. Key Substance Use and Mental Health Indicators in the United States: Results from the 2017 National Survey on Drug Use and Health. Available at: https://www.samhsa.gov/data/sites/default/files/cbhsq-reports/NSDUHFFR2017/NSDUHFFR2017.pdf Last accessed 4 Mar 21. Rockville, MD: Center for Behavioral Health Statistics and Quality, Substance Abuse and Mental Health Services Administration, U.S. Department of Health and Human Services. ; 2017. Contract No.: SMA 18–5068, NSDUH Series H-53.

[41] Manthey J, Shield KD, Rylett M, Hasan OSM, Probst C, Rehm J (2019). Global alcohol exposure between 1990 and 2017 and forecasts until 2030: a modelling study. Lancet.

[42] Gangitano A. Coronavirus brings quick changes to state alcohol laws. The Hill https://thehillcom/business-a-lobbying/business-a-lobbying/490514-coronavirus-brings-quick-changes-to-state-alcohol (Last accessed 1 Mar 2021). 2020.

[43] Rundle-Thiele S, Siemieniako D, Kubacki K, Deshpande S. Benchmarking alcohol literacy: a multi-country study. Mod Manage Rev. 2013;18:99–111.

[44] Buykx P, Gilligan C, Ward B, Kippen R, Chapman K (2015). Public support for alcohol policies associated with knowledge of cancer risk. Int J Drug Policy.

[45] Jernigan D, Ross CS. Supplement: The alcohol marketing landscape: alcohol industry size, structure, strategies, and public health responses. J Stud Alcohol Drugs Sup. 2020;19:13–25. 10.15288/jsads.2020.s19.13 PMC7064002 32079559

