# Supplementary material for: Alcohol outlets and alcohol consumption in changing environments: prevalence and changes over time
Source: Subst Abuse Treat Prev Policy. 2022 Feb 4;17:7. doi: 10.1186/s13011-021-00430-6 (PMC8815126; doi:10.1186/s13011-021-00430-6)
Supplement: Supplementary file 1 — Additional file 1. Supplement to main text description of the analytical sample. Supplement Fig. 1. Enrollment and retention. Supplement Table 1. Baseline policies related to off-premise sale of alcohol in Pennsylvania and the two comparison states (New Jersey and Delaware). Supplement Table 2. Additional details on alcohol consumption measures. Supplement Table 3. Characteristics of included vs. excluded participants. Supplement Table 4. Alcohol consumption and alcohol outlets, baseline and the one-year follow-up for total, Pennsylvania and non-Pennsylvania, N = 772. Supplement Table 5. Sensitivity analysis - sample subset to drinkers. Adjusted cross-sectional estimates of alcohol consumption with density of off-premise outlets and distance to outlets. N = 565. Supplement Table 6. Sensitivity analysis - sample subset to drinkers. Multinomial regression, adjusted within-person change in alcohol consumption (change in days, drinks, binge occasions) for an increase in off-premise alcohol outlets within a 1.6 km buffer (> 0 increase in outlets). N = 521. Supplement Fig. 2 Adjusted cross-sectional estimates of exposure to off premise alcohol outlets (density of and distance to outlets) and number of drinks per day, stratified by gender. [file 13011_2021_430_MOESM1_ESM.pdf]

Supplement File

Title: Alcohol outlets and alcohol consumption in changing environments: prevalence and changes over time

Authors: Amy H. Auchincloss*, Saima Niamatullah, Maura Adams, Steven Melly, Jingjing Li, Mariana Lazo

* Correspondence to: E-mail: aha27@drexel.edu

List of supplementary Figures and Tables

Supplement to main text description of the analytical sample ... 2

Supplement Figure 1. Enrollment and retention ... 2

Supplement Table 1. Baseline policies related to off-premise sale of alcohol in Pennsylvania and the two comparison states (New Jersey and Delaware) ... 3

Supplement Table 2 Additional details on alcohol consumption measures ... 4

Supplement Table 3. Characteristics of included vs. excluded participants ... 5

Supplement Table 4. Alcohol consumption and alcohol outlets, baseline and the one-year follow-up for total, Pennsylvania and non-Pennsylvania, N=772 ... 6

Supplement Table 5. Sensitivity analysis - sample subset to drinkers. Adjusted cross-sectional estimates of alcohol consumption with density of off-premise outlets and distance to outlets. N=565 ... 7

Supplement Table 6. Sensitivity analysis - sample subset to drinkers. Multinomial regression, adjusted within-person change in alcohol consumption (change in days, drinks, binge occasions) for an increase in off-premise alcohol outlets within a 1.6 km buffer (>0 increase in outlets). N=521 ... 8

Supplement Figure 2. Adjusted cross-sectional estimates of exposure to off premise alcohol outlets (density of and distance to outlets) and number of drinks per day, stratified by gender. ... 9

Supplement to main text description of the analytical sample

There were 2555 survey participants aged ⩾21 who were asked the alcohol questions at baseline, and 802 of these participants responded to the follow-up survey (31% retention). Losses to follow-up were primarily due to no response after at least 7 call attempts (50%) or refusal at follow-up (hard refusal, requests to be added to do not call list, or other reason). Among the 802 participants who responded to the follow-up survey, 30 participants were excluded due to missing outcome or exposure data, leaving 772 participants in the cross-sectional analyses.

Despite low cohort retention at follow-up, those included vs. lost-to-follow-up were mostly similar (Supplement Table 1) and the analytic sample was comparable to the age, gender, race distribution in the census population in the areas where the cohort resided (U.S. Census Bureau, 2018). The exception was that the lost-to-follow-up sample had more participants with other race, lower income and lower education, and higher proportion of non-drinking. The lost-to-follow-up pattern was similar for Pennsylvania and non-Pennsylvania except that a larger share of non-Whites were lost-to-follow-up in non-Pennsylvania.

Citation: U.S. Census Bureau. (2018). QuickFacts Available online: https://www.census.gov/quickfacts/fact/table/philadelphiacitypennsylvania,philadelphiacountypennsylvania,philadelphiacitymississippi,US/PST045217. Last accessed Februrary 1, 2019. Retrieved from Washington, DC:

Supplement Figure 1. Enrollment and retention

Baseline survey
Enrolled December 2016-February 2017
N= 2767

Excluded N = 212
Age < 21 not asked alcohol questions

Age >=21
N= 2555

Excluded N= 1753
Did not respond to follow-up survey December 2017-February 2018

Retained in 12-month follow-up survey (December 2017-March 2018)
N= 802

Excluded N= 30
Refused to answer alcohol questions n= 2
No outlet data (followup address unable to be geocoded or no longer lives in PA, NJ, DE) n = 28

Analytic sample: Retained in cross-sectional analyses
N= 772

Excluded N= 58
Only 58 participants experienced a decrease in alcohol outlets at follow-up.
This was too few to operationalize as a discrete exposure category so they were dropped for longitudinal analyses.

Analytic sample: Retained in longitudinal analyses
N= 714

Supplement Table 1. Baseline policies related to off-premise sale of alcohol in Pennsylvania and the two comparison states (New Jersey and Delaware)

State	Pennsylvania, original policies (baseline)		New Jersey	Delaware
	**Liquor and wine**	**Beer and wine sellers**		
**Sales hours**	**Mon to Sat:** 9:00am to 10:00pm **Sunday:** 11:00am - 7:00pm	**Mon to Sat:** 9:00am to 10:00pm **Sunday:** 11:00am - 7:00pm	**Mon to Sat:** 9:00am to 10:00pm **Sunday**: 9:00am to 10:00pm (although some counties have reduced Sunday hours)	**Mon to Sat:** 9:00am to 1:00am **Sunday:** 12:00pm to 8:00pm
**Are there State-run stores?**	Yes, State-run stores for liquor and wine		No State-run stores	No State-run stores
**Liquor**	State-run stores		Liquor stores, private	Liquor stores, private
**Wine**	State-run stores		Liquor stores, private	Liquor stores, private
**Beer**		Beer stores (AKA 'distributors') were generally only permitted to sell cases beer, 12 or 24 beers in each case	Non-chain retail stores. *	Liquor stores, private

* New Jersey (NJ) did not have a ban on selling beer at retail stores. However, NJ limited each company to only two licenses (reference: stateliquorlaws.com/state/NJ).

Supplement Table 2 Additional details on alcohol consumption measures

Supplementary text to main text: Outcomes: Alcohol consumption

Participants aged ⩾21 were asked if they consumed any type of alcoholic beverage in the past 30 days. Only past-30 days was asked in order to keep the phone survey focused/brief (and has been used by others (CDC, 2019)). If the participant answered yes' to past 30 day alcohol consumption, then subsequent questions were asked regarding alcohol consumption.

Subsequent questions were asked regarding the number of days alcohol was consumed ("In the past 30 days, how many days per week or per month did you drink any alcoholic beverages?"), and the number of drinks consumed per day ("Thinking about the most recent occasion when you drank any alcoholic beverage, how many drinks did you have?"). A question was also asked about binge drinking, using the definition from the U.S. Substance Abuse and Mental Health Services Administration (SAMHSA, 2017) as drinking ⩾5 alcoholic drinks for males and ⩾4 for females on a single occasion ("Many people are able to drink X or more alcoholic beverages on an occasion. In the past 30 days, on how many days do you think you had X or more drinks of any alcoholic beverage on an occasion? An example of an occasion would be one evening.") Because Pennsylvania's relaxation of alcohol control primarily increased access to wine and beer, the survey also asked what type of alcohol was consumed in the past 30 days. However, results were not shown by alcohol type because 90% reported consuming both beer and wine and only 10% consumed soley spirits.

Citations:

CDC. 2019 BRFSS Questionnaire https://www.cdc.gov/brfss/questionnaires/pdf-ques/2019-BRFSS-Questionnaire-508.pdf (Last accessed March 20, 2021). 2019.

SAMHSA. Binge Drinking: Terminology and Patterns of Use, 2016. Available at: https://www.samhsa.gov/data/report/2017-methodological-summary-and-definitions. (Last accessed 1/18/17).

Supplement Table 3. Characteristics of included vs. excluded participants

		Participants Included		Participants Excluded (after remove ineligible [ages 18-<21])	
		N	Column %	N	Column %
		772	100%	1783	100%
Age Group				212	
	21 to 34	197	26%	545	31%
	35 to 49	218	28%	518	29%
	50 to 64	357	46%	720	40%
Gender					
	Female	402	52%	949	53%
	Male	370	48%	834	47%
Race					
	Black	259	34%	658	37%
	White	419	54%	754	42%
	Other	94	12%	371	21%
State					
	Pennsylvania	442	57%	963	55%
	Delaware	151	20%	321	18%
	New Jersey	179	23%	470	27%
Consumed alcoholic beverage in past 30 days					
	No	268	35%	726	41%
	Yes	504	65%	1049	59%
		Mean	STD	Mean	STD
Income per capita		$33200	$22100	$31300	$23300
Years of education		14.81	2.34	14.04	2.24

Supplement Table 4. Alcohol consumption and alcohol outlets, at baseline and one-year follow-up for total, Pennsylvania, and non-Pennsylvania, N=772[*]

	Baseline									Followup time period								
	All			Pennsylvania			Non-Pennsylvania			All			Pennsylvania			Non-Pennsylvania		
	N	Col %		N	Row %		N	Row %		N	Col %		N	Row %		N	Row %	
	772			444	58%		328	42%		772	100%		444	58%		328	42%	
**Alcohol consumption**																		
Consumed alcoholic beverage in past 30 days				N	Col %		N	Col %		N	Col %		N	Col %		N	Col %	
No	268	35%		171	39%		97	30%		276	36%		166	37%		110	34%	
Yes	504	65%		273	61%		231	70%		496	64%		278	63%		218	66%	
Higher consumption, binary indicators																		
High alcohol consumption relative to others in cohort (defined as number of drinks per week top quintile, >=8 drinks per week)																		
No	650	84%		376	85%		274	84%		651	84%		371	84%		280	85%	
Yes	122	16%		68	15%		54	16%		121	16%		73	16%		48	15%	
NIAAA, higher risk alcohol use disorder (defined as males>14 drinks/week, females>7 drinks/week)																		
No	697	90%		405	91%		292	89%		686	89%		393	89%		293	89%	
Yes	75	10%		39	9%		36	11%		86	11%		51	11%		35	11%	
Binge drinking in past 30 days (defined as males >=5 drinks, females>=4 drinks)																		
Don't Know/Refused	7	1%		5	1%		2	1%		5	1%		4	1%		1	0%	
No	555	72%		319	72%		236	72%		543	70%		313	70%		230	70%	
Yes	210	27%		120	27%		90	27%		224	29%		127	29%		97	30%	
	Median	P25	P75	Mediar	P25	P75	Median	P25	P75	Median	P25	P75	Median	P25	P75	Mediar	P25	P75
Alcohol days, drinks, binge occasions (continuous)																		
# days consumed alcohol, *per week* [b]	0.75	0	2	0.75	0	2	0.75	0	2	0.75	0	2	0.75	0	2	1	0	2
# drinks, *per week* [b]	1	0	4.5	1	0	4	1	0	5	1	0	4	1	0	4.75	1.5	0	4
# binge occasions, past 30 days	0	0	1	0	0	1	0	0	1	0	0	1	0	0	1	0	0	1
	Median	P25	P75	Mediar	P25	P75	Median	P25	P75	Median	P25	P75	Median	P25	P75	Mediar	P25	P75
**Alcohol outlets**																		
Off-Premise Outlets, network distance to nearest outlet																		
Miles	0.522	0.342	0.942	0.464	0.313	0.686	0.753	0.387	1.408	0.548	0.338	0.962	0.455	0.297	0.693	0.795	0.385	1.523
Meters	835	547	1507	742	500	1098	1205	619	2253	877	541	1539	729	475	1109	1272	616	2436
Off-Premise Outlets, number in buffer																		
1600 meter buffer	5	1	10	7	3	13	2	0	5	5	1	10	7	3	13	2	0.5	5
Off-Premise Outlets, number in buffer, per 10,000 population																		
1600 meter buffer	1.8	0.9	2.9	1.7	1.0	2.6	1.9	0.0	3.4	1.7	1.0	2.8	1.7	1.1	2.7	1.8	0.2	3.3
**Population density**																		
Population density, population in the 1.6 buffer (per 1,000)																		
1600 meter buffer	24.9	9.4	50.3	45.3	24.4	62.6	9.4	6.2	19.4	25.1	9.9	51.7	46.8	24.1	64.5	10.0	6.6	19.6

SD: standard deviation, P25: 25th percentile, P75: 75th percentile, Col = column, NIAAA: U.S. National Institute on Alcohol Abuse and Alcoholism

a Includes participants who did not consume alcohol in past 30 days (N = 207 or 27% of the cohort)

b Note that in main text Table 1, consumption is shown*per month* (whereas here we show consumption *per week*).

Supplement Table 5. Cross-sectional sensitivity analysis - sample subset to drinkers.

Adjusted[a] cross-sectional estimates of alcohol consumption with density of off-premise outlets and distance to outlets. N=565

Exposure			Number of drinking DAYS per week [b] (continuous counts)				Number of DRINKS per week (continuous counts)				HIGH number of drinks per week [c] (binary variable)				BINGE in past 30 days [d] (binary variable)			
			Exp (Beta) [c]	95% CI low	95% CI high	p value	Exp (Beta) [c]	95% CI low	95% CI high	p value	Odds Ratio b	95% CI low	95% CI high	p value	Odds Ratio b	95% CI low	95% CI high	p value
**Alcohol outlet density in 1.6 km buffer, per 10,000 population**																		
Quartiles																		
Q1.	Lowest	0.0 - 0.99	Referent				Referent				Referent				Referent			
Q2.		1.0 - 1.70	1.04	0.86	1.26	0.697	1.08	0.96	1.21	0.205	1.36	0.70	2.65	0.368	1.09	0.65	1.82	0.757
Q3.		1.71 - 2.8	1.17	0.97	1.41	0.096	1.27	1.14	1.42	<.0001	2.20	1.16	4.16	0.016	0.92	0.55	1.55	0.765
Q4.	Highest	2.9 - 10.7	1.26	1.06	1.50	0.008	1.33	1.20	1.48	<.0001	1.61	0.86	3.02	0.141	1.15	0.70	1.90	0.580
**Distance from participant to nearest off-premise outlet**																		
Tertiles (kilometers and miles)																		
T1.	Nearest	0.021 - 0.622 km (0.01-0.386 miles)	Referent				Referent				Referent				Referent			
T2.		0.623 - 1.26 km (0.387-0.78 miles)	1.04	0.89	1.21	0.643	0.98	0.90	1.07	0.692	0.95	0.57	1.61	0.860	1.13	0.72	1.77	0.590
T3.	Farthest	1.27 - 10.16 km (0.79-6.31 miles)	0.84	0.69	1.01	0.069	0.81	0.72	0.91	0.0003	0.61	0.31	1.19	0.146	1.33	0.77	2.30	0.300

a Cross-sectional results follow-up, adjusted for age, sex, race/ethncity, per capita income, educational attainment, history of chronic disease (binary), state.
When per-population was not part of the exposure measure, then the model also adjusted for population density within a 1.6 km area (operationalized into quartiles).
Within each quartile of the standardized count, the median (and p25, p75) of the unstandardized 1.6km density is as follows: Q1: median 0 outlets (0-1); Q2: median 6 outletS (2, 8);; Q3: median 7 outlets (3, 13); Q4: median 11 outlets (4, 25)

b Beta coefficients derived from Poisson regression. Beta represents the difference in the logs of expected drinking days (per month) for discrete exposure category vs. referent level.
Here, exponentiated beta coefficients represents a relative value. Thus, in cross-sectional data the exp(beta) 1.35 can be interpreted
as 35% higher drinking days per month when living in the highest quartile of outlet density (0.29-1.7 per 10,000 population) relative to the lowest quartile (the referent group).

c Odds ratios derived from logistic regression. High consumption relative to others in cohort (top quintile >= 8 drinks per week)

Supplement Table 6. Longitudinal sensitivity analysis - sample subset to drinkers.

Multinomial regression, adjusted[a] within-person change in alcohol consumption (change in days, drinks, binge occassions) for an increase in off-premise alcohol outlets within a 1.6 km buffer (>0 increase in outlets). **N=521** [b]

**Outcome**		N	% in sample	Adjusted Odds Ratio [c]	95% CI low	95% CI high	p value
Change in number of drinking days per week							
0	No change	95	18%	Referent			
1	Increased	206	40%	1.38	0.70	2.73	0.354
2	Decreased	220	42%	1.00	0.51	1.95	0.989
Change in number of drinks per week							
0	No change	46	9%	Referent			
1	Increased	244	47%	0.79	0.34	1.85	0.591
2	Decreased	231	44%	0.48	0.21	1.15	0.099
Change number of binge occasions (past 30 days) [d]							
0	No change	290	56%	Referent			
1	Increased	126	24%	0.96	0.54	1.70	0.885
2	Decreased	105	20%	0.81	0.43	1.51	0.509

a Adjusted for age at baseline, sex, race/ethnicity, per capita income, education, history of chronic disease (binary), moved from ZIP code at followup, state at followup (Pennsylvania vs non-Pennsylvania), and population density per 1.6 km area (quartiles).

b The exposure is a binary variable: increase in outlets vs. no increase in outlets (referent category) using the measure 'count of outlets in 1.6 km buffer'. The category for 'decrease' in outlets was not included because very few participants experienced a decrease in outlets. Per population standardization was not needed for the exposure variable in longitudinal model because the exposure was within-person change in outlet exposure and population density did not change much (because participants remained in their state). Nevertheless, we included population density (quartiles) as an adjustment variable in the model.

c Odds ratios derived from multi-nomial logit regression as appropriate for 3-level outcome: alcohol consumption no change (referent category), decrease, increase. Change defined as |>0| days per week, |>0| drinks per week, |>0| binge days per month.

d Binge refers to past 30 days consumed a large volume of alcohol during a single occasion (>=5 drinks for males, >=4 drinks for females).

Supplement Figure 2. Adjusted cross-sectional estimates of exposure to off premise alcohol outlets (density of and distance to outlets) and number of drinks per day, stratified by gender.

There was no strong evidence that there were cross-sectional differences by gender in the association between alcohol outlets (density or proximity) and drinking days or high alcohol use or binge in past 30 days (P for interaction > 0.09 and no evidence from longitudinal analyses that there were differences by gender in the association between change in outlet and change in consumption (p for interaction > 0.18). The exception to this was in cross-sectional analysis, the association between alcohol outlets (density or proximity) and drinks per week was stronger for males than females (P for interaction <0.0001, Supplement Figure 2). For example, males living in the highest density area (quartile 4 vs. 1) had 35% more drinks per week vs. females 24% more drinks per week (males expβ 1.35, 95% CI: 1.18, 1.54; females expβ 1.24, 95% CI: 1.04, 1.48). The association between living in an area farthest from an alcohol outlet and lower number of drinking days was only apparent for males (tertile 3 vs. 1 males expβ 0.62, 95% CI: 0.53, 0.72). whereas for females the association was positive (which was the inverse of our hypothesis, expβ 1.25, 95% CI 1.03, 1.51).

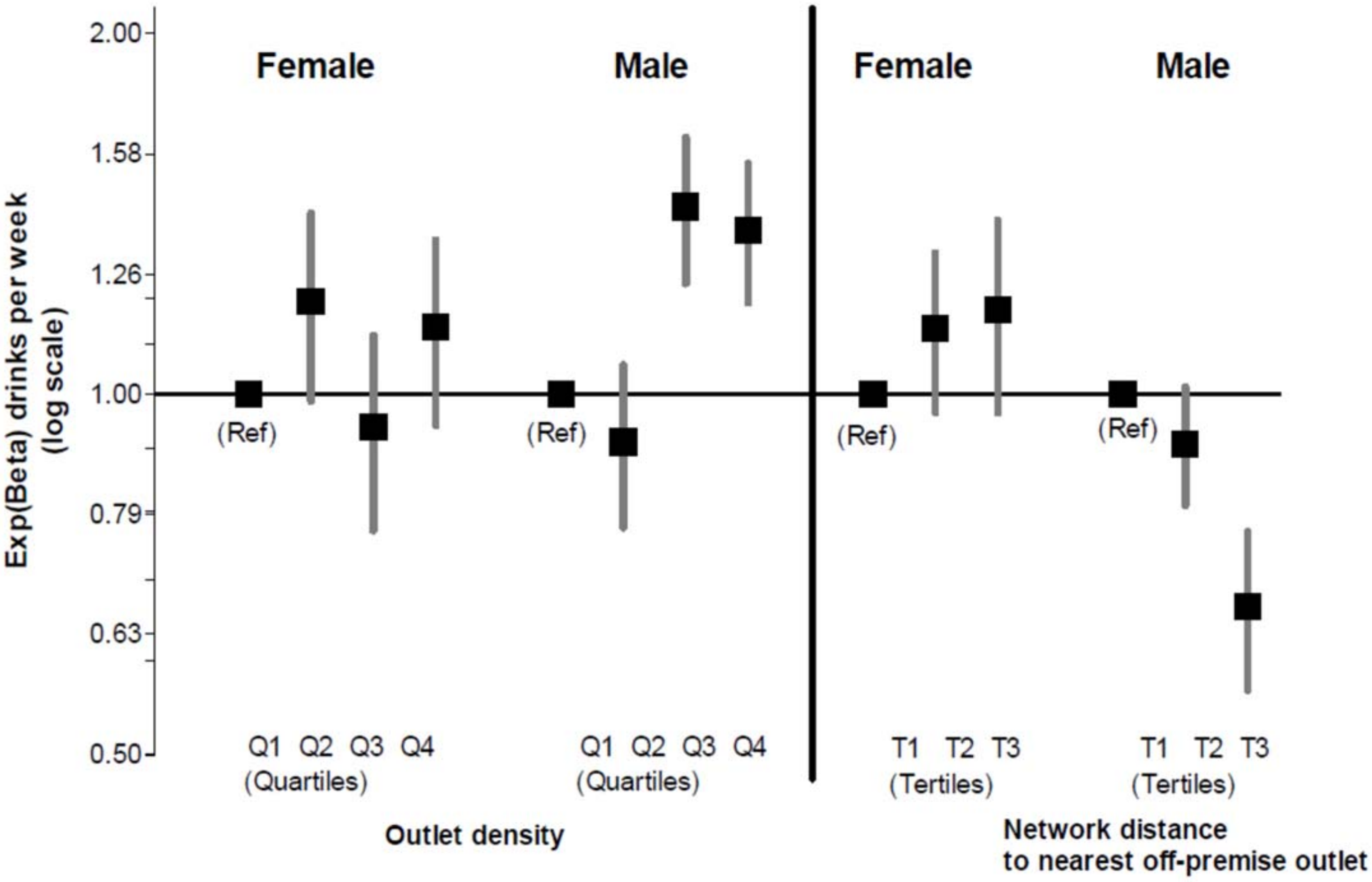

SD: standard deviation, P25: 25th percentile, P75: 75th percentile, Col = column, NIAAA: U.S. National Institute on Alcohol Abuse and Alcoholism

a Includes participants who did not consume alcohol in past 30 days (N = 207 or 27% of the cohort)

b Note that in main text Table 1, consumption is shown per month (whereas here we show consumption per week).
